# The “Cycle” of HIV: Limits of Personal Responsibility in HIV Vulnerability Among Transgender Adolescents and Young Women in Lima, Peru

**DOI:** 10.1007/s10461-024-04462-4

**Published:** 2024-08-22

**Authors:** Casey Orozco-Poore, Amaya Perez-Brumer, Leyla Huerta, Ximena Salazar, Aron Nunez, Africa Nakamura, Rodrigo Aguayo-Romero, Alfonso Silva-Santisteban, Sari L. Reisner

**Affiliations:** 1 Department of Child Neurology, UCLA Mattel Children’s Hospital, University of California Los Angeles, Los Angeles, CA USA; 2 Department of Medicine, Harvard Medical School, Boston, MA USA; 3 Division of Social and Behavioural Health Sciences, Dalla Lana School of Public Health, University of Toronto, Toronto, ON Canada; 4 Feminas Peru, Lima, Peru; 5 Centro de Investigacion Interdisciplinaria en Sexualidad, Sida y Sociedad, Universidad Peruana Cayetano, Lima, Peru; 6 Queens House of Nakamura, Peruvian Kiki Ballroom, Lima, Peru; 7 Division of Endocrinology, Diabetes and Hypertension, Brigham and Women’s Hospital, Boston, MA USA; 8 Department of Epidemiology, University of Michigan School of Public Health, Ann Arbor, MI USA; 9 Department of Epidemiology, Harvard T.H. Chan School of Public Health, Boston, MA USA

**Keywords:** Transgender, Women, HIV, Adolescent, Resilience, Community, Stigma, Latin America

## Abstract

Globally, transgender women (TW) face a high burden of the HIV epidemic. In Peru, HIV prevalence among TW rises at age 25, indicating a need to understand HIV vulnerability as adolescents reach adulthood. The life course of TW is often marked by abuse, discrimination and poverty fueled by transphobic stigma. Approaches to the HIV epidemic among TW and adolescents emphasize problem behaviors such as unprotected sex and substance. However, there has been a call for HIV research and interventions to understand and leverage community strengths. This qualitative study utilized a transgender-oriented, strength-based, intersectional and feminist approach to understand the strengths and protective health behaviors among 17 transgender adolescents and young women (TAYW) age 16–24 in Lima, Peru. Most participants re-located to Lima from the Amazon due to familial rejection, and engaged in obligatory sex work. TAYW demonstrated self-knowledge, motivation for education, efforts to secure employment, strong community networks, legal advocacy, avoiding problem substance use, HIV knowledge and condom use. However, strengths were impeded by multi-level barriers such as familial physical abuse, educational discrimination, and sexual assault which led to increased HIV vulnerability. We created a conceptual model of the “cycle” of HIV to describe the limits of personal responsibility within a vulnerable community denied access to family, education, employment and human rights. We recommend researchers, clinicians and public health workers follow the lead of TAYW at the frontlines of the HIV epidemic, and support beloved communities and enabling environments which may permit protective behaviors to mitigate HIV vulnerability.

## Resumen

A nivel mundial, las mujeres transgénero (MT) enfrentan una alta carga de la epidemia del VIH. En Perú, la prevalencia del VIH entre las MT aumenta a los 25 años, lo que indica la necesidad de comprender la vulnerabilidad al VIH a medida que las adolescentes llegan a la edad adulta. El curso de vida de las MT suele estar marcado por el abuso, la discriminación y la pobreza por culpa del estigma transfóbico. Los enfoques sobre la epidemia del VIH entre las MT y los adolescentes enfatizan conductas problemáticas como el sexo sin protección o el uso de sustancias. Sin embargo, existe un llamado a realizar investigaciones e intervenciones sobre el VIH para comprender y aprovechar las fortalezas de la comunidad. Este estudio cualitativo utilizó un enfoque feminista, interseccional, basado en fortalezas y orientado a las personas transgénero para comprender las fortalezas y los comportamientos protectores de la salud entre 17 adolescentes y mujeres jóvenes transgénero de 16 a 24 años en Lima, Perú. La mayoría de las participantes migraron a Lima desde la Amazonía debido al rechazo familiar y se dedicaron al trabajo sexual obligatorio. Las adolescentes y mujeres jóvenes transgénero demostraron autoconocimiento, motivación para la educación, esfuerzos para conseguir empleo, redes comunitarias sólidas, defensa legal, evitar el uso problemático de sustancias, conocimiento sobre el VIH y uso de condones. Sin embargo, las fortalezas se vieron obstaculizadas por barreras de múltiples niveles, como el abuso físico familiar, la discriminación educativa y la agresión sexual, que llevaron a una mayor vulnerabilidad al VIH. Desarrollamos un modelo conceptual del “ciclo” del VIH para describir los límites de la responsabilidad personal dentro de una comunidad vulnerable a la que se le niega el acceso a la familia, la educación, el empleo y los derechos humanos. Recomendamos que los investigadores, médicos y trabajadores de la salud pública sigan el ejemplo de adolescentes y mujeres jóvenes transgénero en la primera línea de la epidemia del VIH y apoyen a comunidades queridas y entornos propicios que puedan permitir conductas protectoras para mitigar la vulnerabilidad al VIH.

## Introduction

Globally, transgender women (TW) face a significantly higher burden of the HIV epidemic, with an overall HIV prevalence of 19.9% [1] and a risk of HIV acquisition 12 times higher than the general population [2]. In Peru, HIV prevalence among TW ranges from 29.8 to 48.8% [3–5], compared with 0.3% in the general population [6]. As Peruvian transgender adolescents and young women (TAYW) approach the age of 25, HIV prevalence dramatically increases, underscoring the urgent need for a heightened understanding of the vulnerability TAYW experience across the lifespan, to inform HIV prevention and care strategies [4]. A “life course” approach to the health of TAYW, which elucidates how social identity influences health trajectories across developmental periods [7], is a useful tool for considering factors which may worsen or mitigate HIV vulnerability for vulnerable groups such as transgender adolescents reaching adulthood in Peru.

The life course of transgender children often includes physical abuse, neglect, educational discrimination, bullying and stigma due to transgender identity. Research carried out in the United States elucidates that transgender individuals face heightened adverse childhood experiences (ACEs), particularly neglect [8]. ACEs such as sexual abuse, physical violence and neglect have been found to contribute to increased HIV prevalence [9–11]. The U.S. Center for Disease Control reported that 87.4% of LGBTQ students heard negative remarks about transgender people [12], and 35% of transgender youth are bullied at school [13]. The 2015 US Transgender Survey reported that 10% of transgender adults experienced familial physical violence, approximately one third experienced healthcare discrimination, 30% suffered employment discrimination, and almost half were verbally harassed for being transgender in the past year [14]. Homelessness was reported by 30% of participants, 29% were living in poverty, and 12% had exchanged sex for income [14]. Research demonstrates factors such as early educational attrition, unemployment, intimate partner violence, poverty, and housing insecurity are associated with high HIV prevalence, and negatively impact HIV prevention and treatment outcomes [15–20]. Globally, transphobic stigma, which leads to interpersonal, social, and structural violence, fuels HIV risk throughout development [21].

Available literature among adult Peruvian TW similarly reflects a context characterized by marginalization, discrimination, and oppression. Peruvian transgender women who migrate to Lima, the urban capital of Peru, from the Amazon experience stigma throughout migration leading to poverty, violence, exploitation, survival sex work, and related state violence [22]. The Peruvian government maintains a weak legal framework to protect vulnerable transgender people [23] and violent policing of sex workers [24]. Despite biomedical advancements including HIV pre-exposure prophylaxis (PrEP), as of 2023 PrEP is not widely available as a public health policy in Peru despite being available through research studies [25]. A 2012 study found that out of 450 TW in Peru, 64% endorsed sex work as their main source of income [5]. The same study found that among the 335 TW in Peru who endorsed gender-affirming procedures, 40% had injected silicone in the past, introducing a risk of HIV through needle sharing within low-resource contexts without medical support [5]. These existing, albeit limited, data suggest transgender women in Peru experience diminished access to health.

Despite immensely discriminatory contexts, dominant approaches to the global HIV epidemic among transgender women point to “risk behaviors” as causing heightened HIV prevalence [26]. Data collection on adolescents worldwide emphasizes problem behaviors, including the national United States Youth Risk Behavior Surveillance System which “tracks behaviors that can lead to poor health” [27]. The World Health Organization has honed in on unprotected sex, alcohol and drug consumption as causative agents of the HIV epidemic experienced by young transgender people [28]. Research indicates TAYW have lower rates of PrEP uptake and adherence [29]. In Peru, research has similarly identified low utilization of Peruvian public services and HIV testing among transgender women [30]. Substance use, depression, and intimate partner violence were identified as prevalent among TW in Peru, and may contribute to HIV risk [31, 32]. A common thread among most of these data is the measurement of risk behaviors thought to forecast HIV vulnerability. However, disparities in healthcare access can also be contextualized as driven by multi-level barriers including anti-transgender stigma [33, 34], unequal power dynamics within healthcare systems, and limited culturally competent gender minority and youth programs [35, 36].

Recently, there has been a call for HIV prevention, care and research to build on the strengths of individuals and communities vulnerable to HIV to mitigate the HIV epidemic, rather than emphasizing deficits [37, 38]. Strengths-based HIV interventions most often aim to increase the resiliency of individuals or small groups, by fostering characteristics considered to predispose to protective health behaviors [39]. Resilience is described as a “buffer” to adversity, allowing an individual to overcome adversity [39]. Resilience-based HIV research and interventions center on traits considered to be personal strengths such as a self-awareness, sense of self-efficacy, assertiveness, personal competency, and adaptation to adversity [39–43]. A study of Canadian women living with HIV reported that resiliency factors such as personal competence and self-acceptance mediate the relationship between depression and HIV-related medical outcomes [44]. Another less commonly discussed but essential aspect of resilience extends past the individual, to describe community and structural strengths such as activism [38].

More research is needed to understand health promotion strategies among TAYW in global settings outside of the United States and Europe [37]. Existing studies on the health and wellbeing of transgender people identify gender affirmation, social support, and non-traditional healthcare models as protective health strategies [45]. Medical gender affirmation (i.e., feminizing hormones) is associated with positive mental health, HIV prevention, and HIV care outcomes [46–52]. Accessing medical care displays resilience factors such as self-knowledge, and working to attain goals [39–43]. Social gender affirmation such as using a chosen name represents an important health determinant to decrease suicidality among transgender youth [46–48, 53], about which little is known in the Peruvian context. Existing literature on protective mechanisms evidences that Peruvian TW leverage social capital as a protective factor against HIV vulnerability and can increase access to healthcare, social support, and other resources [45]. Group-based behavioral interventions, that leverage the strengths of group dynamics and peer support, have demonstrated improved acceptance, uptake, and adherence to PrEP, while also increasing knowledge about HIV among TAYW aged 16–29 years [54]. Research suggests HIV testing delivered in mobile clinics as opposed to fixed clinics are more likely to reach and newly diagnose HIV among Peruvian TW, likely due to proximity to community hubs of TW and existence outside of discriminatory contexts such as traditional healthcare settings [55]. A 2023 study with 20 Peruvian TW highlights social support as an essential component of adopting health-protective behaviors such as PrEP use [56]. There may be additional unique aspects HIV-protective strengths among young transgender communities in Peru.

Although early data suggest transgender women in Peru experience a high burden of HIV and exercise resilience, there are significant gaps in the literature, particularly regarding the lived realities of made-vulnerable Peruvian TAYW throughout their life course. More research is needed to understand the dynamics of HIV vulnerability, resiliency within adversity, and resistance to adversity among Peruvian TAYW, which may elucidate potential opportunities to increase access to healthcare. There is also a need to understand how social policies impact vulnerability for transgender people, particularly those living in Latin America [57]. Addressing these gaps, we qualitatively explore social context and HIV-related health vulnerabilities, strengths, and protective health behaviors described by TAYW in Lima, Peru. Learning from the lived experiences of TAYW in Lima, this paper aims to describe the unique contextual factors surrounding HIV vulnerabilities at the intersection of gender identity and the adolescent to young adult life course.

## Methods

### Participants and Procedures

Between November 2019 and February 2020, TAYW in Lima, Peru ages 16–24 years (*n* = 17) were interviewed to characterize and understand HIV vulnerability in developmental, psychological, and social contexts. TAYW were purposively sampled [58] for transgender female identity, age, and residency in Lima utilizing in-person and online peer-based recruitment methods. Written informed consent was obtained before the interviews. Interviews lasted 1.5–2 h, were conducted in Spanish by native Spanish-speaking interviewers, and were audio recorded and transcribed verbatim. Institutional Review Board approval was obtained from Mass General Brigham (protocol #2020P003584) and Universidad Peruana Cayetano Heredia (protocol #208049). This study was conducted in collaboration with Féminas, a transgender women’s community organization in Lima, Peru. Part of our study team was hired from Féminas, and study protocol as well as interviews were held at Féminas to meaningfully integrate lived expertise and center participant comfort. A Community Advisory Board was also formed in partnership with trans-led community organizations. Analysis and member checking were facilitated through public conversations (*conversatorios*) with community members within Féminas Peru, and Ballroom Peru.

### Measures

A semi-structured qualitative interview guide was used to assess HIV vulnerability, developmental factors, psychological health, and social contexts to identify exposures related to the HIV epidemic. The domains covered in the qualitative interview guide included social context, mental health, HIV vulnerability, resiliency, sexual behaviors, substance use, gender-affirming healthcare, stigma and discrimination, violent experiences, and social networks. Demographic characteristics were also collected (age, birthplace, education, insurance, living situation, gender identity, and gender-affirming interventions) using a brief written survey.

### Data Analysis

A transgender-oriented [59, 60], strengths-based [38], intersectional [61] and feminist approach [62] was applied to analyze the transcripts. This approach acknowledges transgender identity formation as a strength, identifies goals, competencies, and skills as opposed to deficits and risks [63], recognizes intersectional identities and discrimination, identifies community-level strengths including resistance and advocacy, and creates knowledge from women’s lived experiences [64]. This was done by adapting the immersion crystallization method [65] of analysis to identify themes of strengths and positive behaviors, and outcomes of these efforts throughout childhood, adolescence, and adulthood in accordance with a life course perspective. Further, descriptions of violence, including stigma and discrimination, were analyzed inductively (contextualized within the transcript) and deductively (guided by pre-existing theoretical framework) by utilizing bell hook’s pedagogy of social transformation, which has provided a theoretical roadmap for emancipatory research methods [64]. Our emancipatory analysis coded for contexts which challenged dominant themes of discrimination, such as community solidarity, creation of beloved, safe communities, and other sources of social transformation away from violence [64]. Two team members independently coded transcripts. Themes and initial findings were collaboratively and iteratively discussed and distilled with the larger research team as data analysis progressed. A conceptual model was created in consultation with community partners to create a visual representation of the lived experiences of TAYW, and the developmentally specific exposures that increase vulnerability to HIV.

## Results

### Sample Characteristics

The sample mean age was 21 years (range 16–24 years), with 4 participants under the age of 18. Participants described themselves as a “chica” (girl), “mujer” (woman), “trans femenina” (transfeminine), “femenina” (feminine – adjective and noun), “mujer trans” (trans woman), “chica trans” (trans girl) and “transexual mujer” (transsexual woman). The majority of participants (67%) migrated to Lima from the Amazon, and 85.7% reported their primary source of income was from sex work. The majority (71%) of participants did not have higher education. Most (80%) endorsed hormone use, and 94% reported wanting further gender-affirming surgery.

### The “Cycle” of HIV

Most participants disclosed widespread societal discrimination attributed to being transgender. An 18-year-old summarized: “There are very few people who won’t discriminate against us [trans women]… just because we have a different appearance”. Participants described verbal, physical, sexual, and financial abuse at interpersonal, community, societal, and systemic levels.

Interviews underscore the complexity of HIV vulnerability (i.e., the cycle of HIV), emphasizing the disconnect between protective behaviors and positive health outcomes, namely avoidance of HIV. HIV vulnerability among transgender adolescents and women was described as a “cycle” by a 17-year-old: “The cycle. I mean we all just die because of [HIV]. Anyway, I take care of myself, but I know that obviously I’m going to catch it, right”. This participant expressed the importance but futility of personal responsibility in an environment in which she could not alter her health trajectory due to being a *transfemenina* adolescent.

**Fig. 1:**
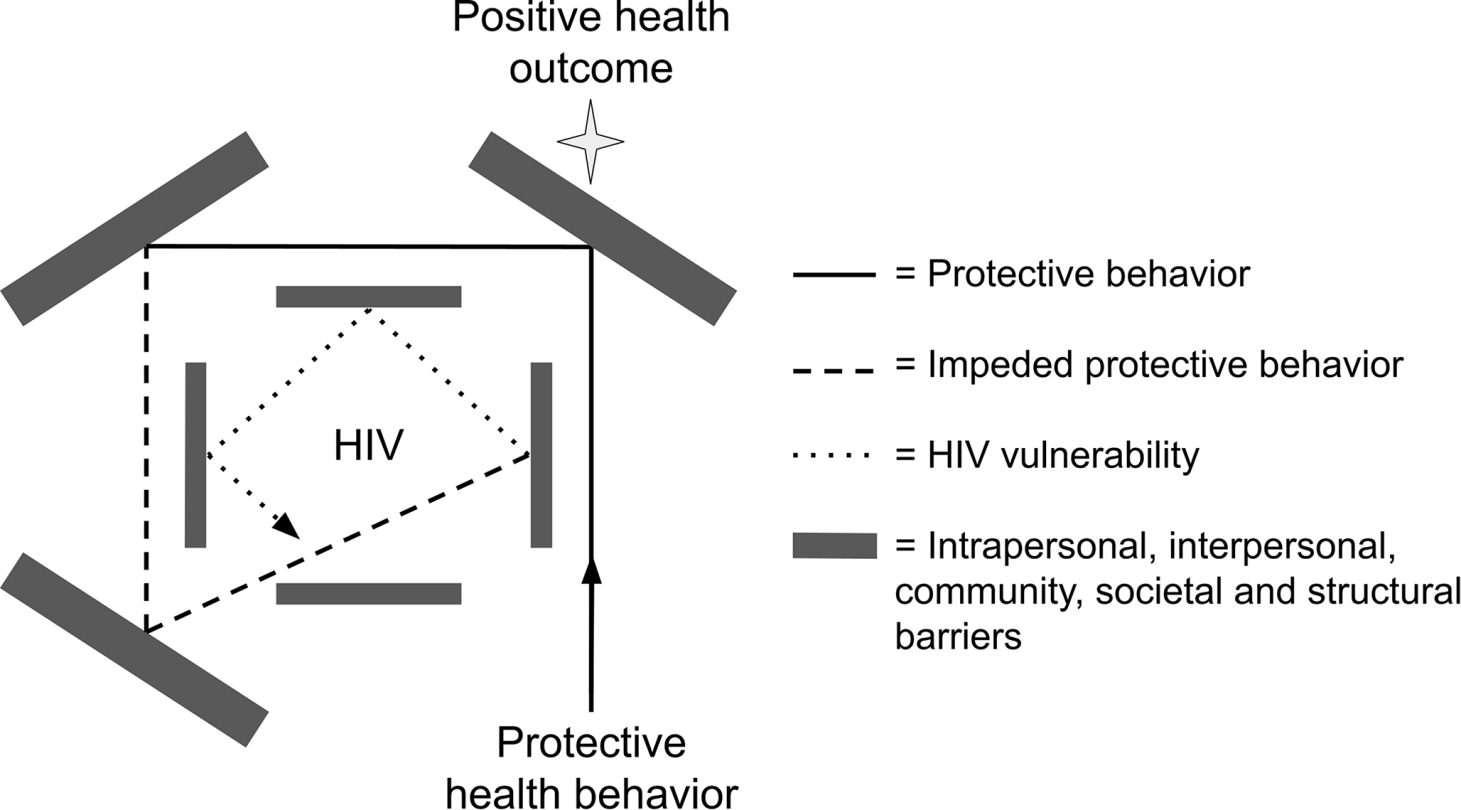
The “Cycle” of HIV: Figure 1 illustrates **protective behaviors** directed towards **positive health outcomes**, which are impeded by intrapersonal, interpersonal, community, societal and structural barriers. These barriers influence and redirect the outcomes of behaviors, and eventually force participants into **HIV vulnerability**. Within the range of HIV vulnerability, individual protective behaviors do not reliably result in protection from HIV. The experience of being forced into HIV vulnerability despite health maintenance was described by a *transfemenina* adolescent as the “cycle” of HIV

The figure displays how a protective health behavior is impeded by multi-level barriers fueled by transphobia and result in HIV vulnerability. Within severely vulnerable contexts such is the case for TAYW in Peru, individual protective behaviors do not reliably result in protection from HIV. Specific examples of the ways that the model can be applied are shown in Table 1 which highlights themes of protective behaviors, associated resiliency, multilevel barriers, and HIV-related health outcomes based on the lived experiences of participants in this study. In the following sections, we uncover how enacted protective behaviors are carried out and thwarted by pervasive multi-level stigma and discrimination (see Fig. 1).

**Table 1:** Stigma and discrimination impede HIV-protective outcomes of resilience and protective health behaviors. Due largely to transphobic stigma and discrimination, the protective health behaviors of TAYW were redirected to health outcomes which contribute to HIV vulnerability

Domains	Resiliency characteristic	Protective health behavior	Multi-level barriers	HIV-related outcome leading to HIV vulnerability
Transgender identity	Self-knowledge, ability to finish goals	Seeking and securing gender affirming interventions	Lack of healthcare access, medical misinformation	Informal silicone injection use
Family	Closeness with family	Efforts to maintain loving relationships	Caregiver violence and rejection	Early homelessness
Education	Self-efficacy, the belief that getting an education is important	Attempts to attend or return to school	Bullying and discipline in school due to transphobia, poverty, lack of familial support	Early academic attrition
Employment	Motivation to secure job skills	Searching for employment	Transphobic discrimination in employment	Unemployment, trafficking, and obligatory sex work
Transgender community	Supporting peers, drawing support from community	Offering emotional and material support	Competition within scarcity	Social isolation
Legal and human rights	Advocating for a better future	Public denouncements	Police violence	Lack of safe pathways to end violence
Substance use	Recreation that does not center drugs / alcohol	Avoiding problem drug / alcohol use	Financial compensation for substance use during obligatory sex work	Sex with substance use
HIV knowledge and practices	Self-advocacy, health maintenance and planning, community connectedness	Community-based HIV knowledge, safer sex practices, inquiring about PrEP / interest in PrEP	Sexual assault and refusal of condoms during sex work, unavailability and misinformation of PrEP	Not taking PrEP, condomless sex

### Domains of Strengths and Protective Health Behaviors within Discriminatory Contexts

In the following sections, we will present the reported and enacted resiliencies, protective behaviors, multi-level barriers and social transformations within the domains of transgender identity, family, education, employment, community, legal protections and human rights, substance use, and healthcare. First, we present descriptions of resilience and enacted protective behaviors. Second, we present how these actions are impeded by intrapersonal, interpersonal, community, societal and structural barriers (see Table 1). Finally, if present in the data, we introduce anecdotes of social transformations which challenge the dominant themes of discrimination.

#### Transgender Identity

##### Self-knowledge and Self-actualization of Transgender Identity

Participants demonstrated strong self-knowledge of femininity and gender identity at young ages. Many stated they always or from birth knew they were *femenina*: “As I always say, I was born that way, since I was little” (21-year-old participant). Participants described themselves working towards becoming “mujeres” (women) at early ages. A 23-year-old participant said, “I knew when I was in kindergarten. I liked acting like a woman… dressing like a girl.” Efforts to align behavior with self-knowledge included “playing house, playing with dolls, jumping ropes, and everything that girls did” (23-year-old). Another 23-year-old participant recounted “I’ve been [femenina] since I was 6, 7 years-old…[my family] had me dance– and I danced well– I put on my mother’s heels and danced.” An additional 23-year-old participant echoed that enjoyable, affirming self-expression was often related to being able to express oneself authentically, particularly to present a *femenina* appearance: “at 14, 15 I already loved everything about women’s clothes. I liked to dress-up, I liked make-up.” These statements reflect high self-understanding of TAYW at early ages, and creative efforts to authentically express oneself.

Participants described clear gender affirmation goals and efforts to secure goals, and most expressed the objective of having a “woman’s body”. From childhood, this very commonly manifested as growing out one’s hair: “I let my hair grow” (23-year-old) and “I grew my hair longer and longer” (23-year-old). Growing out one’s hair was frequently reported as a source of pride and self-expression in childhood. Participants also reported medically supported gender affirmation. An 18-year-old participant described, “I started…taking [estrogen pills], and in that short time my breasts started to grow a little bit. And that made me feel happy.” Other participants desired surgical modifications: “I want surgery on my body” (17-year-old). While some TAYW secured medical prescriptions for hormones, many were not supported by a physician.

##### Obstacles to Transgender Self-actualization

Although in childhood participants described a strong understanding of self which was expressed through dress and hair length, it was often reported that parents and teachers forcibly cut participant’s hair: “I always cried when they cut my hair, I didn’t like when they cut my hair” (18-year-old). This participant describes distress when forced to present in a manner disharmonious with their gender identity.

Despite interest in medically supported gender affirmation, many participants were under-informed about their options to medically transition safely. Participants frequently reported use of silicone injections in domestic settings: “Two girls I paid…they came [to my house] to inject the silicone” (23-year-old). Many lacked access to sterile healthcare settings to medically affirm gender.

Participants reported negative side effects of silicone injections. A young 17-year-old reported: “I fall asleep late, at 4… [to] 6 in the morning. Since I got silicone, I can’t sleep. It’s very uncomfortable, it’s moving… my silicone has slid down.” Other reported negative consequences included pain, itching, and rash.

Many participants described not fully understanding the risks of silicone, and many would not repeat the procedure. A 23-year-old stated, “No, I didn’t know [about the risks]. I wouldn’t inject myself with silicone [again].” This participant went on to express interest in estrogen hormone therapy. This narrative elucidates lack of fully informed consent, and interest in safer alternatives.

#### Relationship with Family

##### Efforts to Retain Relationships: Transgender Identity Concealment

Despite endorsing a strong sense of self and self-efficacy, participants described concerted efforts to hide their identity and gender-related interests from their families. Main motivations for non-disclosure were desire to maintain positive relationships with their family, and fear of abuse. A 19-year-old recounted a shared experience of *femenina* presentation in private, “I used eye shadow, hidden”. Identity concealment was recounted to negatively impact the ability to openly express oneself to others as children. This impeded the progress of early strengths and developing behaviors, such as skills in self-expression and fashion, which were unable to develop publicly or with support from caregivers.

##### Caregiver Violence

When gender identity was disclosed or discovered, often at 10–14 years old, participants endorsed intense surveillance. A 23-year-old reported: “My uncles and aunts…yelled at me…‘how could you be [transgender]’… I wanted to come [to Lima], because I couldn’t leave my house all day…[they] had me under surveillance… kept the front door locked”. Many participants reported similar experiences of constricted autonomy to inhibit authenticity in gender presentation and hobbies.

Most participants described severe physical abuse. A 23-year-old recounts her experiences with domestic abuse: “My parents… hit me with a shovel, when they found out. For being femenina… they threw chili pepper in my eye, I couldn’t see. They hit me with sticks. [From] 10 years old, until I was 12.” Participants often withstood years of abuse aimed at ending *femenina* behaviors.

The majority of participants left home to search for physically and psychologically safe environments during adolescence. An 18-year-old described recounted: “My father didn’t accept me, and it got to the point that had to leave home. I stopped studying, I left… when I was 15… As they say in the Amazon, ‘I went to the city, leaving everything behind’.” Educational attrition was a shared immediate consequence of being forced from one’s childhood home. Across the study sample, TAYW expressed sadness from lack of familial connectedness, and desire to reunite with their childhood community: “I miss my family, and the food I ate over there” (23-year-old). Other participants echoed experiences of ostracization from family and culture, particularly TAYW who had migrated from the Amazon.

##### Family Social Transformations

Although many participants described fleeing their homes due to parental abuse, siblings were often described as providing emotional and material support: “I talk to my sister… she understands” (18-year-old) and “I moved into my brother’s room…I came to [Lima to] study” (23-year-old).

A minority of participants described scenarios of social transformations with caregivers and older relatives toward acceptance of transgender identity, which challenged the dominant arc of caregiver rejection.

A 17-year-old joyfully spoke about her quinceañera, a significant cultural tradition for girls in Peru:

“My 15-year-old dream came true…I had my quinceañera with a dress, everything. In Yurimagua…my homeland…With the support of my family, but not my father. He is very macho… [But] my mother [supported me]… my aunts helped me, I rented a place…friends decorated, there was a DJ. It was beautiful”.

This anecdote describes the powerful potential for the values of family connectedness and cultural tradition to supersede prejudice towards transgender children.

Several participants reported reconnection with family after rejection. A 19-year-old described a restored relationship with family 5 years after leaving the Amazon at 13 years old due to physical abuse:

Last year I visited them…and they accepted me… we talked, we clarified things… how I need them to accept me, as a trans girl, asking that they treat me how I identify. They accepted me, they treated me well, and everything changed. My father…used to not accept me, he beat me, he threatened me…[But then] he sat down, cried and accepted me, [saying] ‘well I can’t do anything about it’.

Acceptance was sometimes attributed to parental acceptance of the permanence of medical or surgical gender transition. These participants expressed gratitude about improved relationships with caregivers.

#### Education

##### Motivation for Education

Many participants described themselves as intelligent, academically motivated students: “School was beautiful, studying…was beautiful…I didn’t care what people said to me. I wanted to learn more and more” (19-year-old). Enthusiasm for education in the face of verbal discouragement reflects a deep sense of self-worth and perseverance.

Participants often elaborated on plans to return to school: “I have a goal… to finish my studies…to study nursing” (23-year-old). Durability of educational aspirations was common, and identified as a hope for the future.

Participants narrated diligent efforts to stay enrolled. A 23-year-old described navigating college and employment: “[I worked] from 6:30 PM to 3:30 AM, then I had school at 6:30 AM. I got up at 5, got ready, caught up…[but] it wasn’t enough time. I tried to do my best to not neglect my studies.” The burden of fully supporting oneself while studying often led to burn out and educational attrition.

##### Educational Exclusion and Bullying

Participants frequently described rejection by academic institutions, teachers and administration. Participants were disciplined for long hair, clothing that did not align with masculine expectations, and name changes. A 17-year-old described their issues with *femenina* presentation: “I went to school with long hair. Eventually…they didn’t let me in… They wanted me to cut my hair”. Fellow participants also reported having their hair forcibly cut at school, or needing to cut their hair to attend school: “If I want to study, I need to cut my hair” (23-year-old). Another 23-year-old reflected on her experience of being forced from school: “When I was in 5th grade, I was already very *femenina*, and… the principal spoke to my mother and they made us transfer schools. I heard him say they were dismissing me because they didn’t allow scandals, for me to be like that [femenina]… it was traumatic… I experienced very discriminatory situations [at school].” These experiences of gender conversion efforts and gender-based discrimination were described as distressing and traumatic, and ultimately prevented access to education.

Peer bullying made school attendance difficult for many participants. A 23-year-old said “Yes [I experienced transphobia]…all year long they bullied me”. Another 23-year-old participant elaborated on the consequences of bullying in her life as a high schooler who “dressed like a woman”. She reported, “At school sometimes there are children who are cruel… they take it out on the students, you know? Yes, I have suffered from bullying, but I have self-respect too… but that [bullying] had a big influence on me not finishing school because they treated me like that… They try to humiliate you.” These participants emphasized the extensiveness of educational discrimination and exclusion, and the relationship between peer bullying and academic attrition. This quote also highlights resilient efforts to retain internal self-respect throughout experiences of external disrespect.

TAYW participants very often shared the sentiment of “I don’t think I will be accepted to study” (21-year-old). Anticipatory stigma informed by prior actualized stigma inhibited school attendance, despite a strong desire for education.

##### Educational Social Transformations

An educational social transformation which deviated from the theme of educational attrition due to discrimination was described by an 18-year-old transgender woman:

“The whole school knew [I was trans], but they loved me…. I dared to say [I was trans] to everyone… at a school-wide event… It started out as a normal speech, but at the end I told them I don’t feel like a man, I feel like a woman, and that I need their support… Tears came to my eyes. I felt inspired, and they all applauded me. I was the most beloved at school. My teachers hugged me and told me… we love you…you can always rely on our support. I was eventually recognized as the valedictorian. They all called me Ivana”.

This rare but powerful anecdote demonstrated the power of academic acceptance of transgender people to facilitate a very successful educational trajectory.

#### Employment

##### Efforts to Secure Employment

Participants widely endorsed a motivation to work and secure job skills: “Really, I love to work” (23-year-old). Another 23-year-old said, “I want to study hairdressing, I want to learn how to cut hair, because I don’t like the streets.” TAYW primarily sought out work in beauty (i.e., clothing, hair, nails) and food services. Several participants also wanted to become nurses or doctors.

During her job search, a 23-year-old participant reported she was told, “You are not going to find work in Lima… Cut your hair, dress like a man. You have silicone, it will be difficult to get a job [looking] like that” to which she responded “But why? I will try”. This demonstrated a tenacity to strive towards employment in the face of immense stigma-related obstacles.

##### Employment Discrimination and Obligatory Sex Work

Many workplace environments explicitly prohibited feminine gender expression at work for individuals assigned male at birth. A 17-year-old stated, “They told me, when I started, ‘That’s not allowed here, you cannot come here dressed as a woman. It’s forbidden’.” As a result of the absence of meaningful educational and employment opportunities, most participants described being forced into obligatory sex work.

A 23-year-old Venezuelan immigrant to Peru stated, “I was desperate, desperate because I couldn’t get a job, and I knew why. I looked for normal jobs. In stores, as a designer. I was never hired, and I said well, if I have to do [sex work], I’m going to do it”. The participant described the common theme of efforts to secure employment outside of sex work, discrimination due to transgender identity, and eventual obligatory transactional sex.

When asked about exchanging sex for income as a 16-year-old, a 19-year-old participant laughed and said, “‘the beaten girl’, when older men want to force you, they force you [to have sex], no? More than anything else…”. This participant described older men engaging in non-consensual child sex trafficking, or the obtaining of a person under 18 for a commercial sex act. Another participant elaborated: “They hung me [by the neck]…They stabbed my [leg]…” (23-year-old). The experience of sexual and physical assault was shared with many other participants, highlighting an extreme lack of bodily autonomy within one of the only contexts which provided transgender women with an income.

##### Employment Social Transformations

Despite the dominant theme of unemployment and exclusion, one participant described a social transformation that helped her develop job skills. This 23-year-old reported her aunt was crucial in supporting her hairdressing aspirations:

“I got into hairdressing… my aunt helped me with what little she had. She enrolled me [into school], bought me scissors, all my supplies. I studied everything for a year and a half… and now I am an expert in many things. I know how to do barber cuts, Californian highlights”.

This anecdote highlights the life-changing role a gender-affirming relative can play in accessing job skills. The participant’s aunt did not reject her career interests due to their *femenina* nature, but instead supported the participant’s developing interests which blossomed into professional job skills.

Strong social networks within contexts of sex work also subverted the dominant discrimination experienced as a survival sex worker, and provided a rare space for transgender young people to express themselves.

“[I like] the communication between girls, I felt like I was with my family, my real family, I felt liberated. I felt that I was in my world, that I could shout to the world who I am, how I feel. I felt at home…I felt that this was mine, my space” (23-year-old).

Other participants shared the sentiment that sex work allowed them to present and express themselves authentically, an experience denied to them in most other contexts.

#### Transgender Community

##### Strong Networks among Transgender Women

Participants described community-based organizations as beloved community. A 17-year-old recounted being invited to a weekly meeting: “She said, ‘do you want to go to the community house? I’ll take you’…I thought it was great. All the trans girls. All of them, beautiful girls. From the first one I met, I liked them all.” Community groups provided spaces for transgender women to connect and befriend one another.

TAYW described relationships with older trans women as maternal: “They treat me very well, as if I were their daughter” (18-year-old). These relationships allowed for the experience of being a “daughter” for individuals who often did not have a relationship with their mothers.

Participants endorsed cooperation and service among transgender women: A 21-year-old elaborated on community support: “If one of our friends gets sick, we support her…even if it is only food, even if it is only one sol [30 cents], medicine even if it is only water, anything we can contribute for the trans girls”. These testimonies highlight the low-resource but high-collaboration context of TAYW.

##### Competition within Scarcity

TAYW did report some degree of interpersonal social conflict, attributed to a sense of competition: “When I didn’t have a [feminine] body, everyone was my friend… but once I had a body, I made more enemies than friends. My body was very good, very pretty and all that, and because of this I made a lot of enemies” (23-year-old). The participant stated she made enemies because of her “pretty” body, alluding to the jealousy that can arise within transgender communities due to disparate access to gender-affirming care. Despite frequent endorsements of community connectedness, having access to a gender-affirming intervention which was not commonly accessible could cause social tension.

#### Legal and Human Rights

##### Legal and Human Rights Advocacy

Community organization meetings were described as opportunities to discuss and protest lack of protections for transgender women: “On Christmas… They talked about discrimination on the streets, and denounced that [the government] doesn’t pay attention to us” (19-year-old). One participant proudly endorsed a shared goal of transgender solidarity: “I would like to promote, support and fight for the rights of all of us [transgender] women” (23-year-old).

Transgender women within community organizations worked together to protect one another from serial abusers, and call for government protections. For example, a 19-year-old described a legal victory: “If the girls don’t pay [the fee to stand for sex work], they are threatened. Some girls were beaten… and I heard that a girl recorded a video… and filed a complaint… and now he is [in] jail”. This successful denouncement effectively removed a serially abusive older man engaged in sex trafficking and physical abuse of sex workers.

##### Lack of Safe Reporting Pathways

Although TAYW worked towards accountability for harm endured by their community, particularly among sex workers, participants did not endorse feeling safe reporting incidents of violence to the police. As one 17-year-old explained, “The police don’t even pay attention to you, if you are [a transgender woman]…they just make fun of you.” The lack of support extended to TAYW by police discouraged reporting of sexual assault and physical violence.

Police officers also arrested TAYW for engaging in sex work: “They don’t let us work… they just take our ID… And they hold us [at a precinct] for a while, and they throw us back out” (19-year-old). Cycles of incarceration were common among participants. Participants also described physical violence and theft from police officers: “Sometimes they beat us. Policemen take our money… shove us… insult us… Once a policeman stole 100 soles… Many of my friends don’t know their rights.” (23-year-old). Police officers often used the lack of legal protections for sex work among Peruvian TW, and lack of legal education, to enact violence upon TAYW and prevent access to safe reporting pathways.

#### Substance Use

##### Avoiding Problem Substance Use

Most participants reported little to no desire for recreational substance use. Participants largely did not endorse a significant personal desire to consume substances recreationally: “No, I don’t let off steam with a drink, and I don’t smoke,” (23-years-old). An 18-year-old participant stated, “Well, I go to parties with my friends. But I only go to dance. Drinking, is not for me. Dancing, yes.” Participants enjoyed dancing but did not actively seek alcohol in social settings.

##### Barriers to Avoiding Substance Use

Despite a frequently stated disinterest in recreational substance use, participants reported reluctantly using substances in the context of sex work.

“I don’t like alcohol, I drink out of obligation. [Clients say] ‘I’ll pay for everything tonight, will you drink with me?’ I say ‘I don’t drink’. At the bar, the disco, I only drink… out of politeness. I take a sip, I throw some out, I drink, I throw some out” (23-year-old).

Other participants endorsed financial compensation for drug use during sex.

“I have had clients [ask me to do drugs], but I charge more. For 40 soles I’m not going to smoke…I prefer to earn a little more if I’m going to damage my little brain cells when I smoke. That’s why I don’t smoke” (21-year-old).

Participants described not wanting to partake in substance use to protect their health. Still, these efforts were impeded by living in a context of poverty within obligatory sex work, which compensated TAYW for using substances. Some participants endorsed taking all the substances offered by clients: “Marijuana, cocaine, everything that the men, what [the clients] bring. There are some drugs that I don’t even know the names of” (19-year-old). TAYW consistently highlighted their use of substances to fulfill expectations at work.

#### HIV Medical Knowledge and Practices

##### HIV Knowledge, Testing, Medication Adherence, Interest in PrEP and Condom Use

Transgender community organizations were often described as a hub for HIV knowledge sharing. An 18-year-old describes her introduction to HIV education:

“[A transgender friend] gave me the location, and I came [to the community organization]. I liked it, and to this day I go. They give me condoms from the government. [Before] I did not know about HIV… I thought [HIV was transmitted through] saliva, because of a kiss. This house helped me a lot. They give a lot of good information.”

Most participants endorsed knowledge of HIV transmission, prevention of HIV with condom use, and the importance of HIV and STI testing.

Participants described going to health visits with friends they met at community meetings: “Sometimes I go with her, we go to the hospital to take tests, and we come back together. [My friend] tells us what time to meet, we meet up, and we go to take our tests and come back together” (21-year-old). Community connectedness positively facilitated access to healthcare services such as HIV/ STI testing.

Participants who were HIV positive disclosed successful medication use verified by biospecimen laboratory data: “My viral load is undetectable….and my CD4 is very high. That’s why I’m thankful to God” (23-year-old). This comment reflects knowledge of the biomedical basis of HIV, and consistent daily efforts to care for one’s health.

Several participants had knowledge about PrEP, and all participants (except for one only interested in natural medicine) were interested in using PrEP when informed about its protective function. Unfortunately, the majority of TAYW reported not knowing how to access PrEP, despite successfully interfacing with the healthcare system for other HIV-related resources such as condoms and testing. A small minority of participants reported taking PrEP: “I’m taking PrEP, I go to my check-ups” (23-years-old), but most participants denied taking PrEP. A 21-year-old participant echoed a common sentiment: “I’ve never tried [PrEP]”.

##### Medication Side Effects, PrEP Misinformation and Condomless Sexual Assault

Medication adherence was sometimes negatively impacted by medication side effects. A 23-year-old recounted her experience as an HIV-positive woman taking antiretrovirals: “Sometimes I don’t want to take them. The days I don’t take my pills… I feel calm. I wake up and I don’t feel like I’m on drugs, I don’t feel drugged. It’s traumatic waking up like that”. Participants associated trauma with feeling “drugged”, which may be related to the use of substances within coercive and violent contexts, such as during obligatory sex work with a client who insists on substance use.

A 23-year-old participant reported being told by a physician they could not take PrEP due to being on estrogen hormone therapy, despite being interested in PrEP: “Yes, I really want to [take PREP], but I have also been told that since I take hormones… it wouldn’t be good for me.” Other participants echoed a lack of evidence-based medical advice, particularly surrounding gender-affirming care, which inhibited access to HIV-related care.

Efforts to sustain condom use in sex work were sometimes described as met with violence which undermined sexual safety: “There are some guys who are bad…they misbehave. [They say] ‘No no no, you’re going to fuck like that, without a condom, it’s better.’ Sometimes they grab you harder, [I say] ‘no, no, let me go…or else I’ll scream’” (23-year-old). Despite efforts to resist condomless sex, this young woman was forced to have sex without a condom, introducing HIV vulnerability despite personal responsibility.

## Discussion

Utilizing an intersectional strength-based analytic approach to identify HIV-related protective behaviors, we examined experiences of HIV vulnerability, psychological health, and social contexts among young transgender adolescents and women aged 16–24. Findings underscore that Peruvian transgender children growing into womanhood demonstrated shared strengths such as self-knowledge, self-efficacy, motivation to secure skills, the ability to finish goals, recreation which does not center substance use, community connectedness, self-advocacy and healthcare planning. These components of resiliency manifested in several domains, including gender affirmation efforts, education, employment, transgender community and medical practices. Interviews highlight the prevalence of enacted protective health behaviors such as HIV knowledge sharing, STI testing, and condom use. Community organizations and solidarity efforts among transgender women were identified as hubs of resource sharing and resistance against the HIV epidemic.

Despite these collective strengths, health-promoting behaviors were bound by pervasive transphobia, which was further magnified by intersectional discrimination against Indigenous migrants from the Amazon, low-income people, non-conforming children, and sex workers. These marginalized social identities and related stigma, discrimination, and oppression negatively influenced health trajectories across development. The dominant theme of abuse across contexts underscores the precarious sociopolitical positionality of Amazonian TAYW. *Transfemenina* childhood in Peru is marked with early familial, educational, employment, and societal rejection that often leads to adolescent sex trafficking. It is within this precarious context that transgender youth are carrying out protective behaviors, such as attempting to enroll into school. Despite diligent efforts in the face of adversity, often these efforts were met with immense multi-level barriers including classmate bullying, expulsion for gender nonconformity, and lack of educational protections for transgender students, which did not permit an individual’s strengths to thrive. For example, despite HIV knowledge, STI testing and condom usage, condomless sexual assault during obligatory sex work circumvents the capacity of TAYW to avoid HIV through personal behavior. We created a conceptual model to illustrate how protective behaviors are continually re-directed towards HIV vulnerability. TAYW narratives highlighted how transphobic stigma leading to precarity interferes with protective health behaviors, suggesting limits of personal responsibility in escaping HIV vulnerability.

The classic minority stress model proposes that stigmatized minorities may engage in harmful behaviors which result in adverse health outcomes due to the stigma they experience [66]. Newer models posit resiliency helps buffer against this risk by laying the groundwork for “adaptive” behavioral responses to adversity [67]. However, often a well-meaning emphasis on resilience has paradoxically centered risk, with HIV resilience interventions seeking to mitigate “risky sexual behavior” (e.g., condomless sex, substance use around sex, multiple sexual partners) and augment “resilient sexual behavior” of using condoms, abstaining from substance use around sex, testing for STIs, being adherent to treatment, and advancing HIV knowledge [39, 68, 69]. In both risk and resiliency models, there is an assumption that behaviors lead to outcomes, and if one enacts protective behaviors they will be rewarded with healthy outcomes.

Efforts to increase HIV resilience and behavioral responsibility among cisgender women in the US and in low-income countries have not demonstrated a significant improvement in health outcomes [69–73]. This may, in part, be due to the over-emphasis on personal behavior to overcome adversity, rather than addressing the unique contexts in which individuals enact protective behaviors throughout the life course. There may be a non-linear relationship between behavior and outcome in contexts continually impeded by multi-level barriers. Our findings suggest that for behavioral responsibility to improve the lives of individuals, the individual may need to act within a context that allows for autonomy and agency, rather than a context which denies housing, education, employment, government protections and cultural connection. Individual-level interventions will likely continue to fail to end the HIV epidemic without meaningful societal and structural change.

### Future Directions

There is interpersonal, familial, community, societal and structural work that must be done to remove the multi-level barriers created to harm and exclude transgender women. There are glimpses of a future without the cycle of HIV in anecdotes of transgender acceptance; an aunt buying hairdressing scissors for her transfemenina niece, a mother honoring her transgender daughter with a quinceañera, and a school that recognized a transgender woman as a valedictorian. These social transformations away from discrimination towards what bell hooks described as “beloved communities” [74] highlight the potential to transform people, societies, and systems. Future transformative potentials include family education about gender diversity, legalizing sex work for transgender women in Peru, and laws against anti-transgender educational and employment discrimination. These structural interventions may help facilitate what Shannon et al. described as “enabling environments” for HIV prevention within vulnerable communities, such as women in survival sex work [75]. Indeed, a systematic review elucidated a relationship between sexual minority human rights and HIV prevention success among transgender women [76].

Whereas intersectional, multi-level discrimination constricts TAYW’s positive health behaviors into vulnerability, intersectional identity expands out of vulnerability. The Black feminist concept of “dual vision” describes the heightened ability of those with multiple oppressed identities to perceive society [64]. This concept underscores the leading role of those with intersecting identities such as Latin American, Indigenous, low-income and young transgender women in ending the cycle of HIV. A Canadian study demonstrated transgender women living with and affected by HIV desired enhanced support for pre-existing community organizations to facilitate housing, food, education, job training and mental health initiatives [77]. Outside of academic literature, there is documentation of Peruvian grassroots transgender community organizations such as Féminas Peru mobilizing to feed 200 + transgender people living in Lima during the COVID pandemic [78], and arts ballroom spaces growing transgender community networks and fundraising for youth living with HIV [79]. Our study echoes these strong community networks among TAYW living in Lima, strongholds of HIV knowledge sharing and mutual aid. Working with transgender adolescents and young women as respected experts in HIV prevention could facilitate culturally relevant and community-based HIV interventions.

### Limitations

Several limitations necessitate consideration in this study. Although the study enrolled TAYW ages 16–24 years, most of the sample were over the age of 18. Therefore, experiences described herein may be more generalizable to TAYW in the 18–24 age-range. Further, although Amazonian Indigenous heritage was described by participants and the research team included Afro-Peruvians, this qualitative study did not sufficiently represent the experiences of Black Peruvians. Communities which center Black transgender leadership include the Peruvian ballroom community, an emerging space of resilience and resistance which warrants further understanding and support. Additionally, the identification of health behaviors and barriers was based on self-report, not active data collection at the time of behavioral action. This may result in overestimating prevalence of positive health behaviors due to social desirability bias or recall bias.

## Conclusion

This community-based qualitative study elucidated tremendous strengths and protective behaviors among transgender adolescents and young women, despite neglect and violence. However, the intersectional nature of discrimination and related oppression that transgender women experience in migratory, Latin American, low-income contexts created multiplying barriers preventing protective behaviors from leading to positive health outcomes. Despite this, there were durable examples of community efforts and social transformations away from discrimination, which allowed participants to access education, employment and medical care, helping mitigate HIV vulnerability. We recommend researchers and clinicians center transgender adolescents and young women as leaders in global HIV interventions, and bolster pre-existing community-led efforts to break the cycle of HIV.
