# Correction: The “Cycle” of HIV: Limits of Personal Responsibility in HIV Vulnerability among Transgender Adolescents and Young Women in Lima, Peru

**DOI:** 10.1007/s10461-024-04507-8

**Published:** 2024-09-23

**Authors:** Casey Orozco-Poore, Amaya Perez-Brumer, Leyla Huerta, Ximena Salazar, Aron Nunez, Africa Nakamura, Rodrigo Aguayo-Romero, Alfonso Silva-Santisteban, Sari L. Reisner

**Affiliations:** 1 Department of Child Neurology, UCLA Mattel Children’s Hospital, University of California Los Angeles, Los Angeles, CA USA; 2 Department of Medicine, Harvard Medical School, Boston, MA USA; 3 Division of Social and Behavioural Health Sciences, Dalla Lana School of Public Health, University of Toronto, Toronto, ON Canada; 4 Centro de Investigacion Interdisciplinaria en Sexualidad, Sida y Sociedad, Universidad Peruana Cayetano, Lima, Peru; 5 Feminas Peru, Lima, Peru; 6 Queens House of Nakamura, Peruvian Kiki Ballroom, Lima, Peru; 7 Division of Endocrinology, Diabetes and Hypertension, Brigham and Women’s Hospital, Boston, MA USA; 8 Department of Epidemiology, University of Michigan School of Public Health, Ann Arbor, MI USA; 9 Department of Epidemiology, Harvard T.H. Chan School of Public Health, Boston, MA USA

**Correction: AIDS and Behavior**

10.1007/s10461-024-04462-4

In this Article the Affiliation Details for Authors has been Updated as Follows:

For Author “Amaya Perez-Brumer” were incorrectly given as ‘ Department of Medicine, Harvard Medical School, Boston, MA, USA ’ but should have been “Division of Social and Behavioural Health Sciences, Dalla Lana School of Public Health, University of Toronto, Toronto, ON, Canada” and “Centro de Investigacion Interdisciplinaria en Sexualidad, Sida y Sociedad, Universidad Peruana Cayetano, Lima, Peru”.

For Author “Leyla Huerta” were incorrectly given as “Division of Social and Behavioural Health Sciences, Dalla Lana School of Public Health, University of Toronto, Toronto, ON, Canada” but should have been “Feminas Peru, Lima, Peru”.

For Author “Aron Nunez” were incorrectly given as “Feminas Peru, Lima, Peru” and “Centro de Investigacion Interdisciplinaria en Sexualidad, Sida y Sociedad, Universidad Peruana Cayetano, Lima, Peru” but should have been “Centro de Investigacion Interdisciplinaria en Sexualidad, Sida y Sociedad, Universidad Peruana Cayetano, Lima, Peru”.

For Author “Africa Nakamura” were incorrectly given as “Division of Social and Behavioural Health Sciences, Dalla Lana School of Public Health, University of Toronto, Toronto, ON, Canada” and “Queens House of Nakamura, Peruvian Kiki Ballroom, Lima, Peru” but should have been “Queens House of Nakamura, Peruvian Kiki Ballroom, Lima, Peru”.

